# The relationship between physical exercise and social trust: evidence from the CGSS 2023 and the potential roles of social interaction, social support and class identity

**DOI:** 10.3389/fpsyg.2026.1893211

**Published:** 2026-07-17

**Authors:** Bingyang Zhai

**Affiliations:** College of Physical Education, Kunsan National University, Gunsan, Jeollabuk-do, Republic of Korea

**Keywords:** Chinese general social survey, class identity, physical exercise, psychological effects, social interaction, social support, social trust

## Abstract

**Introduction:**

Physical exercise (PE) may play an important role in enhancing residents’ social trust; however, further research is needed to clarify the mechanisms underlying this association. This study examines the relationship between PE and social trust among Chinese residents and explores its potential mediating mechanisms, thereby providing empirical evidence for understanding the social psychological effects of exercise and promoting social harmony.

**Methods:**

This cross-sectional observational study used data from the nationally representative China General Social Survey (CGSS) 2023. After sample screening, 5,256 valid observations were retained. Multiple linear regression, ordered probit models, propensity score matching (PSM), and stepwise regression, were employed to examine the association between PE and social trust and to identify its underlying mechanisms.

**Results:**

The results showed that PE was positively associated with social trust among Chinese residents. This association varied across population subgroups, with stronger positive associations observed among female residents, young residents, and residents with a university education. Further analysis indicated that social interaction, social support, and class identity partially explained the association between PE and social trust.

**Conclusion:**

This study found that PE was positively associated with social trust among Chinese residents, and that social interaction, social support, and class identity may serve as potential mediating pathways in this association. These findings suggest that community-based PE may be considered a relevant public activity context for understanding residents’ social trust, and may provide empirical reference for designing inclusive, group-oriented physical activity programs in which social trust is taken into account as an important social outcome.

## 1 Introduction

Social trust (ST) refers to individuals’ generalized evaluation of the trustworthiness, benevolence, and behavioral reliability of strangers or members of society in general. As a key socio-psychological foundation, ST plays an important role in sustaining social integration, facilitating public cooperation, and improving the effectiveness of social governance (Zmerli and Newton, 2008; Falcone and Castelfranchi, 2001). In contemporary China, profound socioeconomic transformation has been accompanied by income inequality, social stratification, weakened community ties, and uncertainty regarding social mobility. These structural changes may reduce individuals’ positive expectations of others and social relationships, increase the vulnerability of ST, and pose challenges to social governance and economic development (Ma and Christensen, 2019; Yang and Xin, 2020). Against this background, identifying everyday social resources that may enhance residents’ ST has become an important theoretical and practical concern in the context of social governance modernization and high-quality economic development.

Physical exercise (PE) is a common, accessible, and socially interactive daily activity. It contributes not only to individuals’ physical and mental health (Asztalos et al., 2010), but also to their participation in public life, expansion of social relationships, and acquisition of social support (Schüttoff et al., 2018). In many PE settings, individuals interact with others through shared participation, adherence to rules, cooperation and competition, emotional communication, and mutual assistance. These repeated and rule-guided interactions may strengthen individuals’ perceptions of others’ reliability, enhance their confidence in the stability of social relationships, and reinforce their belief in the orderly functioning of society (Wijndaele et al., 2007; Zhou and Kaplanidou, 2018). Therefore, PE may provide an important everyday context in which ST is formed and reinforced.

However, existing studies have primarily focused on the health-promoting and well-being-enhancing effects of PE (Downward and Rasciute, 2011; Liao et al., 2023; Wang et al., 2026), whereas its association with ST has received comparatively limited attention. More importantly, the potential explanatory mechanisms underlying this association remain insufficiently clarified. Although previous research suggests that PE may be related to individuals’ social psychological perceptions through social interaction, social support, and identity formation (Skinner et al., 2008), it remains unclear whether these factors can explain the association between PE and ST within an integrated analytical framework. In particular, the roles of social interaction, social support, and class identity have not been sufficiently examined as potential pathways linking PE to ST. This gap limits a deeper understanding of the social function of PE and weakens its theoretical explanatory power in social governance research.

Based on this research background and these gaps, this study uses data from the 2023 Chinese General Social Survey (CGSS) and employs ordinary least squares (OLS) regression models to examine the relationship between PE and residents’ ST. Specifically, this study addresses three research questions. First, is PE significantly and positively associated with residents’ ST? Second, does this association vary across different population subgroups? Third, do social interaction, social support, and class identity serve as potential explanatory pathways linking PE to ST?

This study contributes to the literature in three ways. First, by examining the PE–ST relationship using large-scale national survey data, it provides empirical evidence on the social value of PE and extends the dominant research focus from health promotion and well-being enhancement to social integration. Second, by incorporating social interaction, social support, and class identity into the explanatory framework, this study examines whether these factors partially account for the association between PE and ST, thereby advancing a mechanism-based understanding of the social psychological implications of PE. Third, by conducting heterogeneity analyses and multiple robustness checks, this study enhances the reliability of the empirical findings and provides more nuanced evidence for understanding the relationship between PE and ST.

### 1.1 The relationship between physical exercise and social trust

A substantial body of literature has demonstrated that PE serves both health-promoting and social-interaction functions. Beyond improving physical health, PE may also influence social cognition, interpersonal relationships, and behavioral tendencies. In PE settings, participants often experience enjoyment and achievement through coordinated interaction with others while also learning to follow rules, maintain order, and engage in normative cooperation Zhang (2016). These experiences may reduce psychological barriers to interpersonal communication and help individuals develop more positive evaluations of others’ willingness to cooperate, behavioral predictability, and trustworthiness. Consistent with this view, Sobhani and Hashemi (2017) emphasized that sport-for-all activities play important social roles and are closely related to the formation of social trust. Empirical evidence further supports this association. Di Bartolomeo and Papa (2019) found that short-term exposure to physical activity increased individuals’ trust and prosocial behavior in interactive situations, with effects extending beyond the immediate response. Similarly, Qu and Dong (2017) showed that participation in PE effectively improved college students’ interpersonal communication ability and trust. Based on the above, we propose the following hypothesis:

*H1*: PE is positively associated with Chinese residents’ social trust.

### 1.2 Heterogeneous effects of physical exercise on social trust

The mechanisms underlying the formation of ST may vary by gender, which may further shape the association between PE and ST. Previous research has shown that men exhibit higher initial trust toward unfamiliar others than women; however, in unfair interaction contexts, men also show a more pronounced age-related decline in trust (Lemmers-Jansen et al., 2017; Wang and Yamagishi, 2005). In addition, women tend to rely more on relational bases of trust, such as close relationships, emotional bonds, and interpersonal reciprocity, whereas men are more likely to rely on collective bases of trust, including group affiliation, shared identity, and collective membership (Maddux and Brewer, 2005). Because PE is often embedded in interpersonal and group-based contexts, these gendered bases of trust may influence how PE-related interactions are translated into broader social trust. These findings suggest that gender differences may exist not only in levels of trust, but also in the bases of trust and patterns of trust adjustment. Accordingly, we propose the following hypothesis:

*H2a*: The association between PE and residents’ ST differs by gender.

Age-related patterns of ST are heterogeneous. Experimental evidence indicates that trust increases from childhood to early adulthood and then remains relatively stable across adulthood, whereas trustworthiness tends to increase with age (Sutter and Kocher, 2007; Abdelzadeh and Lundberg, 2017). Older adults may also show reduced sensitivity to cues of untrustworthiness, which can lead to greater trust toward potentially untrustworthy others (Castle et al., 2012). In the Chinese context, social trust has been reported to follow a U-shaped pattern across age groups (Gao and Ma, 2025). These findings suggest that ST may differ across life-course stages because of age-related differences in social experience, interaction networks, and risk perception. Such differences may further influence how individuals at different ages perceive, interpret, and internalize the ST generated through PE. Accordingly, we propose the following hypothesis:

*H2b*: The association between PE and residents’ST differs by age.

Education is an important determinant of ST. Previous studies suggest that education broadens individuals’ cognitive horizons and fosters more open, inclusive, and optimistic social attitudes, which are associated with greater trust in others (Borgonovi, 2012; Bjørnskov, 2007; Sturgis et al., 2010). Empirical evidence has generally shown a positive association between educational attainment and ST (Hooghe et al., 2012). However, this relationship is not uniform. Some studies report only a limited effect of education on ST (Oskarsson et al., 2017), In addition, higher educational attainment may increase risk awareness and critical judgment, making individuals more cautious in social interactions and potentially reducing generalized trust (Wu and Shi, 2020). These mixed findings suggest that education may shape both the level and formation mechanisms of ST. Therefore, individuals with different levels of educational attainment may differ in how they interpret the rules, cooperation, andreciprocity embedded in PE, thereby leading to variations in the PE–ST association. Accordingly, we propose the following hypothesis:

*H2c*: The association between PE and residents’ ST differs by educational attainment.

### 1.3 Hypothesis on the mediating mechanism of physical exercise on social trust

Social capital, as an important social resource, is embedded in social participation and interpersonal networks. It encompasses increased social interaction, enhanced social support, and more positive evaluations of one’s social position (Dinda, 2008). Existing studies suggest that social capital may be related to ST by increasing interpersonal familiarity and behavioral predictability, strengthening positive expectations toward others, and enhancing individuals’ sense of social belonging (Song, 2025; Paxton, 1999). Within this framework, PE, as a socially embedded activity in everyday life, may be indirectly associated with ST by reinforcing individuals’ social capital.

Previous studies have suggested that stable and frequent social interaction can strengthen interpersonal familiarity through contact, communication, cooperation, and reciprocal experiences, thereby reducing uncertainty and psychological defensiveness in social relationships and fostering more positive judgments about others’ reliability, goodwill, and behavioral predictability (Singh, 2012; van den Bos et al., 2012). PE also has a strong social dimension, providing a continuous, stable, and low-threshold context for interpersonal interaction (Tao et al., 2025). In group-based physical activities, shared participation, rule compliance, teamwork, and emotional communication may increase opportunities for interpersonal contact, expand social networks, and enhance familiarity and reciprocal experience (Bian, 2020). These factors may be associated with higher levels of ST. Thus, social interaction may constitute an important pathway through which PE is linked to ST. Based on the above analysis, we propose the following hypothesis:

*H3a*: Social interaction mediates the relationship between physical exercise and residents’ social trust.

Social support is an important psychological and social resource that individuals obtain from their social networks, mainly including emotional comfort, instrumental assistance, informational support, and a sense of security (Gottlieb and Bergen, 2010; Yan et al., 2025). Previous studies have shown that care, assistance, and recognition from others can enhance individuals’ social adaptability, improve their evaluations of interpersonal relationships and the social environment, and further shape their judgments about others’ goodwill, reliability, and willingness to cooperate, thereby serving as an important basis for ST (Liu et al., 2019; Guo, 2017). PE is often embedded in activities shared with friends, colleagues, neighbors, family members, or community members. In such contexts, participants may receive support related to exercise skills, health knowledge, and activity information, as well as encouragement, companionship, practical help, and emotional recognition. Thus, PE provides a concrete practical setting in which individuals can perceive social support (Christensen et al., 2006; Sheridan et al., 2014), which may foster more positive interpersonal expectations and higher levels of ST. Therefore, PE may influence ST through social support. Based on the above analysis, we propose the following hypothesis:

*H3b*: Social support mediates the relationship between physical exercise and residents’ social trust.

Class identity is an important socio-psychological factor associated with social trust. Individuals with a stronger sense of class identity are more likely to hold positive evaluations of their social position, perceived social value, and future development opportunities, which may contribute to more favorable interpersonal expectations and social perceptions (Savage et al., 2001). Previous studies have suggested that subjective class evaluation and class identity are positively related to social trust, indicating that individuals who perceive themselves as occupying a higher or more stable social position tend to report higher levels of trust (Güth et al., 2008; Delhey and Newton, 2005). In the context of PE, especially group-based physical activities, participants may develop positive self-evaluations through improved physical fitness, skill acquisition, and sporting achievements, while also accumulating social capital through sustained interpersonal interactions (Fang and An, 2024). These experiences may enhance perceived social recognition, social inclusion, and confidence in one’s social position, thereby strengthening class identity and ultimately contribute to higher levels of social trust. Based on the above analysis, we propose the following hypothesis:

*H3c*: Class identity mediates the relationship between physical exercise and residents’ social trust.

**Figure 1:**
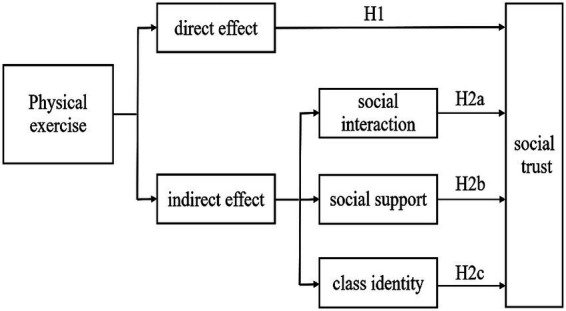
Theoretical model.

Figure 1 presents the theoretical framework linking PE and ST.

## 2 Materials and methods

### 2.1 Sampling

The data used in this study were obtained from the CGSS 2023. Launched in 2003, the CGSS is a nationwide survey of residents in mainland China. It systematically collects multi-level data on society, communities, households, and individuals to document structural changes, social behaviors, and individual perceptions during China’s social transformation. The CGSS employs a sampling design that combines multistage stratified sampling with probability proportional to size sampling, thereby ensuring strong national representativeness and substantial academic value.

Given that the CGSS 2023 is among the most recent publicly available datasets, it provides timely evidence on the social behaviors and psychological perceptions of Chinese residents. Therefore, this study used data from the CGSS 2023 for empirical analysis. The 2023 CGSS covered 31 provincial-level administrative regions in mainland China and collected questionnaire data from 11,326 respondents. The survey included both telephone interviews and face-to-face household interviews. However, the item used to measure generalized social trust, ‘In general, do you think that most people in this society can be trusted?’, was administered only in the face-to-face household interview module. Therefore, the analytical sample was first restricted to respondents who participated in the household survey and answered this module, yielding 5,583 respondents. After excluding 327 observations with missing values in key variables, invalid responses such as ‘do not know,’ ‘unclear,’ and ‘refuse to answer,’ the final analytical sample comprised 5,256 valid observations. Table 1 presents the descriptive statistics of the sample. All data processing and statistical analyses were conducted using Stata 15.

**Table 1:** Demographic characteristics of the sample (*N* = 5,256).

Variable	Category	Frequency	Percentage
Gender	Male	2,351	44.73
	Female	2,905	55.27
Age(years old)	Youth (18–34)	1716	32.65
	Middle-aged adults (35–64)	2,807	53.41
	Elderly (≥65)	733	13.95
Nation	Han nationality	4,773	90.81
	National minority	483	9.19
Individual annual income (yuan)	Low income (≤10,000)	1,101	20.59
	Middle income (10001–29,999)	3,287	62.54
	High income (> 29,999)	868	16.51
Household registration	Rural	2,217	42.18
	Urban	3,039	57.82
Marital status	Married	3,800	72.30
	Unmarried and other	1,456	27.70
Educational attainment	Primary school and below	1811	34.46
	Junior high school	1,534	29.19
	Senior high school	983	18.70
	University	881	16.76
	Graduate studies and above	47	0.89
Religious belief	No religious belief	4,832	91.93
	Has religious belief	424	8.07

### 2.2 Measurements

#### 2.2.1 Dependent variable

ST was used as the dependent variable in this study. It was measured using the item, “In general, do you think that most people in this society can be trusted?” Responses were coded on a five-point scale ranging from 1 = “very distrustful” to 5 = “very trustful,” with higher scores indicating higher levels of social trust. It should be acknowledged that social trust can be conceptualized more broadly, including generalized trust, particularized trust, and institutional trust. However, the CGSS 2023 does not provide a multidimensional trust scale covering these different components. Therefore, this study uses the above single item as a proxy indicator of generalized social trust. Because a single-item measure does not allow for the assessment of internal consistency reliability and may contain measurement error, the findings should be interpreted as associations concerning generalized social trust rather than the full multidimensional construct of social trust. This conceptual and measurement limitation is further discussed in the Limitations section.

#### 2.2.2 Independent variable

SP was used as the independent variable. It was measured using the item, “In the past year, how often did you participate in physical exercise during your leisure time?” Responses were coded on a five-point scale: 1 = “never,” 2 = “several times a year or less,” 3 = “several times a month,” 4 = “several times a week,” and 5 = “every day.” Higher scores indicated more frequent participation in physical exercise.

#### 2.2.3 Mediator variables

Three mediating variables were included in this study: social interaction, social support, and class identity. Social interaction was measured using the CGSS item, “In the past year, how often did you get together with friends in your spare time?” Responses were coded on a five-point scale ranging from 1 = “never” to 5 = “every day,” with higher scores indicating more frequent social interaction.

Social support was measured using the item: “Do you agree that neighbors are willing to help when you are in need?” Responses were coded on a six-point scale: 1 = strongly disagree, 2 = disagree, 3 = somewhat disagree, 4 = somewhat agree, 5 = agree, and 6 = strongly agree. A higher score indicated a higher level of perceived social support. Because a multidimensional scale for social support is unavailable in the CGSS 2023, this study uses respondents’ perceptions of whether neighbors are willing to help when needed as a proxy for support-related assistance and opportunities for neighborhood support exchange. This proxy captures the assistance-related facet of social support rather than its full quality or type, such as emotional support and instrumental support, and does not cover family, friendship, or formal support.

Class identity was measured using the item: “Overall, which social class do you think you belong to in the current society?” Responses ranged from 1 to 10. A higher score indicated a higher self-rated social position. In this study, this variable was used to reflect respondents’ subjective class identity.

#### 2.2.4 Control variables

Following previous studies and considering the availability of variables in the CGSS dataset, this study included several sociodemographic characteristics as control variables, namely gender, age, ethnicity, marital status, household registration status, educational attainment, personal annual income, and religious belief (Xu et al., 2023; Han and Zhao, 2021; Zhai et al., 2026).

### 2.3 Data analysis

#### 2.3.1 Ordinary least-squares (OLS) model

To estimate the association between PE and ST, we specified the following baseline ordinary least squares (OLS) regression model (Equation 1):

$$ST_{i}=\beta_{0}+\beta_{1}PE_{i}+\beta_{2}C_{i}+\varepsilon_{i}$$ (1)

Where $ST_{i}$ denotes the level of social trust of the i-th resident; $PE_{i}$ represents the physical exercise participation of the i-th resident; $C_{i}$ denotes the control variables; $\beta_{0}$ , $\beta_{1}$ and $\beta_{2}$ represent the parameters to be estimated; and $\varepsilon_{i}$ denotes the random error term.

Although ST was measured using a five-point ordinal scale, OLS regression was used as the primary analytical approach because the outcome contains multiple ordered categories and can be treated as approximately continuous in large-scale survey analysis. The OLS model provides directly interpretable estimates of the average association between PE and ST and facilitates comparison across baseline regression, heterogeneity analysis, PSM analysis, and mediation analysis. In addition, Ordered Probit and Ordered Logit models were employed as robustness checks to examine whether the findings were sensitive to the ordinal specification of the dependent variable.

#### 2.3.2 Ordered probit model

Given the ordered categorical nature of ST, estimating it as a continuous variable may overlook its ordinal structure. Therefore, alongside the baseline OLS model, we further specified an ordered Probit model (Equation 2) to examine:

$$ST_{i}^{*}=\alpha_{0}+\alpha_{1}PE_{i}+\alpha_{2}C_{i}+\varepsilon_{i}$$ (2)

where $ST_{i}^{*}$ denotes the latent variable underlying the social trust level of of the i-th resident; $PE_{i}$ is the core explanatory variable, representing the level of physical exercise participation of of the i-th resident; $C_{i}$ denotes the control variables; $\alpha_{0}$ is the constant term; $\alpha_{1}$ captures the estimated association between PE and latent ST; $\alpha_{2}$ denotes the coefficient vector for the control variables; and $\varepsilon_{i}$ is the random error term.

Because the observed ST variable is classified into five ordered categories, four threshold parameters, $\mu_{1}$ , $\mu_{2}$ , $\mu_{3}$ , and $\mu_{4}$ , were introduced to describe the mapping between the latent social trust variable $ST_{i}^{*}$ and the observed social trust category $ST_{i}$ as shown in Equation 3:

$$\begin{array}{c} ST_{i}=\{\begin{array}{cc} 1, & ST_{i}^{*}\leq \mu_{1}\\ 2, & \mu_{1}<ST_{i}^{*}\leq \mu_{2}\\ 3, & \mu_{2}<ST_{i}^{*}\leq \mu_{3}\\ 4, & \mu_{3}<ST_{i}^{*}\leq \mu_{4}\\ 5, & ST_{i}^{*}>\mu_{4} \end{array} \end{array}$$ (3)

Where $\mu_{1}<\mu_{2}<\mu_{3}<\mu_{4}$ When the latent social trust level $ST_{i}^{*}$ crosses different threshold values, individuals are classified into the corresponding observed categories of ST. Since higher values of the observed ST variable indicate higher levels of social trust, a significantly positive $\alpha_{1}$ suggests that higher PE participation is associated with a higher latent level of ST. Conversely, a significantly negative $\alpha_{1}$ indicates that higher PE participation is associated with a lower latent level of ST.

#### 2.3.3 Mediation effect model

We constructed the following mediation effect model (Equations 4 and 5):

$$\begin{array}{c} M_{i}=\rho_{0}+\rho_{1}PE_{i}+\rho_{2}C_{i}+\varepsilon_{i} \end{array}$$ (4)

$$\begin{array}{c} ST_{i}=\partial_{0}+\partial_{1}PE_{i}+\partial_{2}M_{i}+\partial_{3}C_{i}+\varepsilon_{i} \end{array}$$ (5)

Where $M_{i}$ represents the mediating variable for respondent i , including social interaction, social support, and class identity. $\rho_{1}$ indicates the estimated association between PE and the mediating variable, while $\partial_{2}$ captures the association between the mediating variable and ST. $\partial_{1}$ represents the direct association between PE and ST after accounting for the mediating variable. The product $\rho_{1}\times \partial_{2}$ denotes the indirect effect of PE on ST through the mediating pathway. The definitions of the other variables are consistent with those in Equation 1.

It should also be noted that all analyses were conducted using the unweighted analytical sample. Although the CGSS adopts a complex sampling design, the official sampling weight variable applicable to the restricted analytical sample was not available in the data file used for this study. Therefore, sampling weights were not applied in the present study. This may affect the representativeness of the estimates and the validity of population-level inferences. Accordingly, the results should be interpreted primarily as associations observed within the analytical sample rather than as fully weighted population-level estimates.

## 3 Results and analysis

### 3.1 Multiple regression analysis

As shown in Table 2, the coefficient of PE is positive and statistically significant at the 1% level when ST is modeled either as a continuous variable using ordinary least squares (OLS) regression (Column 1) or as an ordered categorical variable using an ordered Probit model (Column 3). After controlling for relevant control variables in Columns 2 and 4, the coefficient of PE remains positive and statistically significant at the 1% level. This pattern indicates that the positive association between PE and ST persists after accounting for a broad set of control variables. Although statistically significant, the magnitude of the estimated effect is relatively modest. Given that PE is a low-threshold, low-cost, and widely practiced form of social participation, even a modest association may have certain practical relevance at the population level, but the effect should not be overstated, thereby supporting H1.

**Table 2:** Multiple regression results.

Variables	ST			
	OLS model		Ordered Probit model	
	(1)	(2)	(3)	(4)
PE	0.094^***^	0.061^***^	0.169^***^	0.113^***^
Gender		0.098^***^		0.181^***^
Age		−0.175^***^		−0.323^***^
Nation		0.079^***^		0.144^***^
Individual annual income		−1.112^***^		−.0207^***^
Household registration		0.080^***^		0.148^***^
Marital status		0.177		0.033
Educational attainment		0.115^***^		0.213^***^
Religious belief		0.059^*^		0.110^*^
Constant	2.728	2.659		
N	5,256			

ST, social trust; PE, Physical Exercise. ^*^p < 0.1, ^**^p < 0.05, ^***^p < 0.01.

### 3.2 Robustness test

To evaluate the reliability and robustness of the multiple regression results, we conducted a series of robustness checks, as reported in Table 3. First, we modified the model specification by re-estimating the association between PE and ST using an ordered Logit model (Columns 1 and 2). Second, we recoded the dependent variable and applied alternative estimation methods. Specifically, responses of “trust” and “very trust” were coded as 1, while responses of “neutral,” “distrust,” and “very distrust” were coded as 0. Logit and Probit models were then used for the regression analysis (Columns 3—6). Third, we included additional control variables in the model, including province, political affiliation, number of family members, and number of properties, and re-estimated the OLS regression (Column 7). Across all specifications, the estimated coefficients of PE remained positive and statistically significant at the 1% level, providing further support for the robustness of the baseline results.

**Table 3:** Robustness test results.

Variables	ST						
	Ordered logit model		Logit model		Probit model		Add control variables
	(1)	(2)	(3)	(4)	(5)	(6)	(7)
PE	0.312 ^***^	0.212 ^***^	0.315^***^	0.217^***^	0.178^***^	0.122^***^	0.059^***^
Constant			−2.464	−2.295	−1.445	−1.337	2.643
Control covariates	NO	YES	NO	YES	NO	YES	
N	5,256						5,229

ST, social trust; PE, Physical Exercise. ^*^p < 0.1, ^**^p < 0.05, ^***^p < 0.01.

### 3.3 Sensitivity test

To control for potential selection bias and examine the sensitivity of the baseline results, this study employed propensity score matching (PSM) to re-estimate the association between PE and residents’ST. Specifically, respondents who reported “never” participating in physical exercise were assigned to the control group and coded as 0, whereas those reporting any level of physical exercise participation were assigned to the treatment group and coded as 1. This dichotomization was used only for the PSM analysis because conventional PSM requires a binary treatment indicator. The analysis compared respondents with any PE participation to those with no PE participation to test whether the baseline association remained robust after reducing covariate imbalance. The original PE variable was retained in the main regression analyses. However, this dichotomization reduces the variability of the original PE measure and cannot capture dose–response differences across PE frequency levels. Therefore, the PSM results should be interpreted as evidence of the association between any PE participation and ST, rather than differences across PE frequency levels.

Based on this classification, three matching approaches were applied: nearest-neighbor matching (k = 2), radius matching (caliper = 0.01), and kernel matching. As reported in Table 4, the estimated matched differences were 0.128, 0.139, and 0.136, respectively, and all were statistically significant at the 1% level. The PSM results were consistent with the baseline regression results, suggesting that the positive association between PE and ST remained after reducing imbalance in observed covariates, which is consistent with Hypothesis H1. However, these results should not be interpreted as causal evidence, because PSM cannot address unobserved confounding and the data remain cross-sectional.

**Table 4:** PSM results.

Matching methods	Treated	Controls	ATT	S.E.	*t*
Nearest neighbor matching (k = 2)	3.137	3.009	0.128^***^	0.021	6.07
Radius matching (caliper = 0.1)	3.137	2.998	0.139^***^	0.020	7.12
Kernel matching	3.137	3.002	0.136^***^	0.019	7.08

The variable for participation in physical exercise has been recoded here, converting it from a five-point variable to a two-point variable. Due to space constraints, the covariate-balance tables for the matched samples are not reported herein. ^***^p < 0.01.

### 3.4 Heterogeneity test

#### 3.4.1 Gender heterogeneity

As shown in Table 5, PE was positively and significantly associated with ST among both male and female residents at the 1% significance level. The estimated coefficient was slightly larger for female residents than for male residents (0.065 > 0.054), suggesting that the association between PE and ST may be stronger among females. This finding indicates gender heterogeneity in the association between PE and ST, thereby supporting Hypothesis H2a.

**Table 5:** Heterogeneity test results of gender.

Variables	ST	
	Male	Female
PE	0.054^***^	0.065^***^
Constant	2.807	2.804
Control covariates	YES	
adj.*R*^2^	0.077	0.080
*N*	2,351	2,905

ST, social trust; PE, Physical Exercise. ^*^p < 0.1, ^**^p < 0.05, ^***^p < 0.01.

#### 3.4.2 Age heterogeneity

As shown in Table 6, PE was positively and significantly associated with ST among youth, middle-aged adults, and elderly residents, with all coefficients significant at the 1% level. The estimated coefficients were 0.089, 0.046, and 0.056, respectively. Although a positive association was observed across all age groups, the coefficient was relatively larger among youth residents than among elderly and middle-aged residents. These results indicate age heterogeneity in the association between PE and residents’ ST, thereby supporting Hypothesis H2b.

**Table 6:** Heterogeneity test results of age.

Variables	ST		
	Youth	Middle-aged adults	Elderly
PE	0.089^***^	0.046^*^	0.056^***^
Constant	2.417	2.375	2.201
Control covariates	YES		
adj.*R*^2^	0.064	0.070	0.086
*N*	1716	2,807	733

ST, social trust; PE, Physical Exercise. ^*^p < 0.1, ^**^ *p* < 0.05, ^***^p < 0.01.

#### 3.4.3 Educational heterogeneity

As shown in Table 7, the association between PE and ST varied across educational groups. Specifically, PE was positively and significantly associated with ST among Chinese residents with primary school education or below, junior high school education, senior high school education, and university education, all at the 1% significance level. The estimated coefficients, ordered from lowest to highest magnitude, were as follows: junior high school education (0.050), primary school education or below (0.052), senior high school education (0.073), and university education (0.075). Although the coefficient for residents with graduate studies or above education was larger in magnitude (0.136), it was not statistically significant. These findings suggest educational heterogeneity in the association between PE and ST. Accordingly, Hypothesis H2c is supported.

**Table 7:** Heterogeneity test results of educational attainment.

Variables	ST				
	Primary school and below	Junior high school	Senior high school	University	Graduate studies and above
PE	0.052^***^	0.050^***^	0.073^***^	0.075^***^	0.136
Constant	2.730	2.850	3.204	3.015	4.610
Control covariates	YES				
adj.*R*^2^	0.060	0.074	0.093	0.081	0.318
N	1811	1,534	983	881	47

ST, social trust; PE, Physical Exercise. ^*^p < 0.1, ^**^ *p* < 0.05, ^***^p < 0.01.

### 3.5 Mediation model results

The stepwise regression method was used to examine the mediating mechanisms underlying the association between PE and ST (Table 8). Column 1 of Table 9 shows that, after controlling for control variables, PE was positively and significantly associated with ST. This result is consistent with the multiple regression findings reported in Column 2 of Table 2, indicating a significant positive association between PE and residents’ ST.

**Table 8:** The mediating effect of PE on ST.

Variables	ST	Social interaction	ST	Social support	ST	Class identity	ST
	(1)	(2)	(3)	(4)	(5)	(6)	(7)
PE	0.061^***^	0.250^***^	0.047^***^	0.087^***^	0.053^***^	0.125^***^	0.043^***^
Social interaction			0.056^***^				
Social support					0.091^***^		
Class identity							0.137^***^
Constant	2.659	2.497	2.519	3.549	2.332	5.503	1.905
Control covariates	YES						
adj.*R*^2^	0.083	0.055	0.096	0.003	0.141	0.003	0.457
*N*	5,256						

ST, social trust; PE, Physical Exercise. ^***^p < 0.01.

**Table 9:** Bootstrap test and decomposition of mediating effects.

Mediating path	Total effect	Direct effect	Indirect effect	Bootstrap SE	95% CI	Mediation proportion
PE → Social interaction → ST	0.061	0.047	0.014	0.002	[0.011, 0.017]	22.95%
PE → Social support → ST	0.061	0.053	0.008	0.002	[0.005, 0.011]	13.12%
PE → Class identity → ST	0.061	0.043	0.017	0.004	[0.009, 0.025]	28.07%

ST, social trust; PE, Physical Exercise.

Columns 2 and 3 of Table 8 report the mediation test for social interaction. The results show that PE was positively and significantly associated with social interaction, and social interaction was also positively and significantly associated with ST. After social interaction was included in the model, the coefficient of PE decreased from 0.061 to 0.047 but remained significant at the 1% level. From the perspective of social interaction, participation in physical exercise may increase opportunities for interpersonal contact, communication, and shared activity, thereby fostering reciprocal social relations and generalized trust (Alesina and La Ferrara, 2002; Eime et al., 2013). Accordingly, the results were consistent with the view that social interaction may statistically account for part of the association between PE and ST; thus, H3a was supported.

Columns 4 and 5 of Table 8 report the mediation test for social support. The results show that PE was positively and significantly associated with social support, and social support was also positively and significantly associated with ST. After social support was included in the model, the coefficient of PE decreased from 0.061 to 0.053 and remained statistically significant. In terms of social support, physical exercise participation may expand individuals’ supportive social networks and increase access to emotional, informational, or instrumental support (Christensen et al., 2006; Golaszewski et al., 2022). Prior research suggests that social support can strengthen positive expectations of interpersonal reliability and reciprocity, which are closely related to higher levels of social trust (Liu et al., 2019; Guo, 2017). Accordingly, the results were consistent with the view that social support may statistically account for part of the association between PE and ST; thus, H3b was supported.

Columns 6 and 7 of Table 8 report the mediation test for class identity. The results show that PE was positively and significantly associated with class identity, and class identity was also positively and significantly associated with ST. After class identity was included in the model, the coefficient of PE decreased from 0.061 to 0.043 but remained significant at the 1% level. For class identity, PE may be associated with stronger subjective class identification by enhancing social participation, self-perception, and perceived social integration (Fang and An., 2024; Hoye et al., 2015). Building on prior research, subjective class identity is closely related to individuals’ sense of social belonging and confidence in social relations, which may further contribute to social trust (Delhey and Newton, 2003). Accordingly, the results were consistent with the view that class identity may statistically account for part of the association between PE and ST; thus, H3c was supported.

To further test the significance of the mediating effects, this study conducted a bootstrap analysis with 5,000 resamples (Table 9). The results show that the indirect effects of social interaction, social support, and class identity were 0.014, 0.008, and 0.017, respectively, accounting for 22.95, 13.12, and 28.07% of the total effect. The 95% confidence intervals for all three indirect effects did not include zero, indicating that these mediating effects were statistically significant. These findings provide further support for H3a, H3b, and H3c.

## 4 Discussion

Using data from CGSS 2023, this study investigated the association between PE and residents’ ST, examined whether social interaction, social support, and class identity were statistically consistent with potential explanatory pathways, and further assessed heterogeneity across gender, age, and educational attainment. In the following section, we provide a detailed interpretation of the main findings.

### 4.1 The impact of physical exercise on social trust

The regression results showed a significant positive association between PE and ST among Chinese residents, which is consistent with previous findings (Di Bartolomeo and Papa, 2019; Zheng et al., 2021). However, the magnitude of this association was relatively modest. Given the large sample size, statistical significance should not be overinterpreted as evidence of a strong substantive effect. Rather, the finding suggests that PE may be associated with a small but meaningful increase in ST. Because PE is a low-threshold, low-cost, and widely practiced form of social participation, even a modest association may have practical relevance at the population level, particularly when PE is integrated into community-based programs and public sports services.”

This positive association may be understood through several possible mechanisms. First, prior studies suggest that physical activity may be associated with lower stress, stronger self-efficacy, more positive emotions, and greater psychological resilience (Singh et al., 2023; Qiu et al., 2025). These psychological resources may help individuals evaluate social relationships in a more open and positive manner, which may be related to higher levels of social trust (Robbins, 2016). Second, PE may provide accessible and relatively stable spaces for public interaction. In such settings, shared participation, repeated interaction, and emotional communication may increase exposure to reciprocal and cooperative social relations, thereby offering a possible context for the development of social trust (Eime et al., 2013; Eather et al., 2023). Third, PE often involves competition, cooperation, and mutual assistance within shared rules. Existing research suggests that such experiences may strengthen perceptions of others’ predictability and reliability, which are conceptually related to social trust (Skinner et al., 2008), which are significant mediators of ST. However, because the present study is based on cross-sectional data, these interpretations should be understood as possible explanatory pathways rather than confirmed causal mechanisms.

### 4.2 Heterogeneity in social trust

It should be noted that the subgroup analyses in this study were exploratory and descriptive in nature. Therefore, differences in coefficient size or statistical significance across subgroups should be interpreted as indications of possible heterogeneity rather than as conclusive evidence of statistically significant between-group variation. The following discussion thus focuses on possible explanations for within-subgroup associations and provides a basis for future moderation analyses.

The subgroup analysis suggested a possible gender-related pattern in the association between PE and ST. The estimated association was positive among both men and women, but appeared descriptively stronger among women. This pattern may be related to gender differences in the relational benefits associated with PE. For women, PE may provide more opportunities for emotional communication, sharing life experiences, and mutual support, thereby enhancing interpersonal familiarity, reciprocity, and emotional connectedness (Oliveira et al., 2014). These relational experiences may facilitate the extension of trust from specific exercise partners to broader social relationships (Niu et al., 2025; Golaszewski et al., 2022). By contrast, men’s participation in PE may be more strongly related to athletic performance, skill improvement, or functional engagement, and may therefore involve different forms of social interaction and support in physical activity contexts (Deaner et al., 2016; Egli et al., 2011). As a result, the spillover from PE-related interpersonal trust to generalized social trust may be more limited among men (Molanorouzi et al., 2015). Thus, the relatively weaker PE–ST association among men may reflect differences in the relational forms and social meanings of PE participation.

The subgroup analysis also indicated a possible age-related pattern, with a relatively stronger estimated association observed among young residents. Young people are at an important stage of social relationship expansion and social adaptation (Kirwan et al., 2025), and PE may provide a meaningful setting for entering groups and building peer relationships (Smith, 2003). Through peer interaction, cooperative experiences, and emotional communication, PE may be more closely associated with ST among young residents. Among elderly adults, PE may contribute to social trust mainly by providing emotional support and reducing loneliness (Gu et al., 2024). However, because older adults’ social networks may be relatively stable, the association between PE and ST may be less pronounced. For middle-aged adults, work, family responsibilities, income pressure, child education, and elder care may constrain the frequency and continuity of PE participation (Weiss et al., 2022; Huxhold et al., 2022). In addition, career development, income pressure, child education, and elder care may reduce discretionary time and constrain the frequency and continuity of PE participation (Venn and Strazdins, 2017). which may limit the extent to which PE contributes to broader social interaction and trust formation. These factors may partly explain why the PE–ST association appeared less pronounced among middle-aged and elderly adults.

The subgroup analysis further suggested a possible education-related pattern, with a relatively stronger estimated association observed among residents with a university education. University education may enhance cognitive ability, social adaptability, and awareness of public participation, enabling individuals to better understand and internalize the rules, cooperation, reciprocity, and emotional communication embedded in PE (Huang et al., 2011). In this context, PE may serve as an important social setting for expanding interpersonal relationships and strengthening social identification, which may help explain the relatively stronger PE–ST association among residents with a university education (Eather et al., 2023). Among residents with graduate education or above, heavier academic or occupational demands may constrain regular participation in PE (Jett et al., 2024), thereby weakening its association with ST. However, the non-significant association in this subgroup should be interpreted with particular caution. Because the number of respondents with postgraduate education or above was relatively small, the statistical power of the analysis may have been limited. Therefore, this result should not be taken as evidence that PE is unrelated to ST in this group. Taken together, these exploratory patterns may reflect differences in cognitive resources, social participation opportunities, time availability, competing academic or occupational demands, and subgroup sample size, and should be further examined using formal moderation models with sufficient statistical power.

Taken together, the heterogeneity analysis provides exploratory evidence of possible subgroup variation in the association between PE and ST. However, because formal interaction tests were not conducted, the observed differences in subgroup-specific estimates should not be interpreted as definitive evidence of statistically significant differences between groups. Therefore, the above discussion should be understood as a cautious interpretation of observed subgroup patterns rather than as formal confirmation of gender-, age-, or education-based differences.

### 4.3 The mechanism of physical exercise affecting social trust

The mediation analysis suggested that social interaction was statistically consistent with a potential explanatory pathway linking PE to ST among Chinese residents. This finding is consistent with previous research showing that PE can promote social interaction (Tao et al., 2025; Kim et al., 2021). PE provides regular and structured opportunities for individuals to participate in shared activities, communicate with others, and cooperate in relatively open social settings. Through repeated interaction, individuals may expand their interpersonal networks, increase familiarity with others, and reduce the uncertainty often associated with weak social ties (Eime et al., 2013; Hoye et al., 2015). Moreover, frequent participation in PE may expose individuals to norms of reciprocity, mutual adjustment, and rule-based cooperation, thereby strengthening their sense of social connectedness (Glanville et al., 2013). Therefore, social interaction may serve as a relational pathway through which PE is associated with higher levels of ST.

Social support was also statistically consistent with a potential explanatory pathway linking PE to ST. PE often takes place in community exercise spaces, shared leisure settings, or group-based activities, where participants may receive emotional encouragement, practical assistance, companionship, and informational support from peers, neighbors, or community members (King et al., 2013). These supportive experiences may enhance individuals’ perceptions that others are caring, dependable, and willing to provide help, thereby reducing interpersonal vigilance and strengthening positive expectations of social relationships (Liu et al., 2019). In turn, higher perceived social support may reinforce a sense of belonging and psychological security, encouraging more favorable evaluations of other people and society more broadly (Tseng, 2023). This interpretation is consistent with related studies showing that social support is closely linked to trust formation and positive social perceptions (Sánchez-Santos et al., 2024; Zhang, 2024).

Class identity was further statistically consistent with a potential explanatory pathway linking PE to ST among Chinese residents. From the perspective of reference group processes in class identity research (Kelley and Evans, 1995), PE, especially group-based physical activity, may provide individuals with opportunities for self-presentation, competence development, and social recognition in a relatively equal and participatory context. Through repeated participation, individuals may improve their physical competence, acquire exercise-related knowledge and skills, and gain recognition from others, which may contribute to more positive evaluations of their social position and personal value (Fang and An, 2024). These positive self-evaluations may strengthen class identity by enhancing individuals’ confidence in their social status, sense of social participation, and perceived inclusion within the broader social structure. Stronger class identity may further promote more positive perceptions of social mobility, fairness, and social order (Wei and Zhang, 2020). In group-based PE, shared rules and task allocation may also reinforce experiences of equality, cooperation, and recognition (Silva et al., 2013; Kim et al., 2022), thereby reducing feelings of relative deprivation and social alienation. Accordingly, class identity may represent a status-evaluation pathway through which PE is associated with higher levels of ST.

Overall, these findings should be understood as potential explanatory pathways that are statistically consistent with mediation, rather than as evidence of causal mediation mechanisms. Because PE, the mediator variables, and ST were measured at the same time point, the temporal ordering among these variables cannot be established in the present cross-sectional study.

Although social interaction, social support, and class identity were examined separately in the empirical analysis, they should not be interpreted as fully independent mechanisms. In real social contexts, PE may increase interpersonal interaction, which can facilitate supportive relationships and strengthen individuals’ sense of social belonging and subjective class identity. Therefore, these mediators may reflect interrelated psychosocial pathways rather than mutually exclusive processes. The potential overlap among these pathways should be further examined in future research.

### 4.4 Implications

Based on the above findings and discussion, we propose three recommendations.

First, PE may be promoted as an accessible public activity that supports social interaction and trust-related social resources. Governments and communities should improve public sports facilities, provide low-threshold exercise opportunities, and create stable spaces for interaction, cooperation, and emotional communication. Given the modest effect size observed in this study, the practical value of PE should be understood in terms of its cumulative and population-level contribution rather than as a large individual-level effect.

Second, PE programs should be designed to strengthen social interaction, social support, and class identity. Communities can develop neighborhood exercise groups, sports clubs, volunteer-led fitness activities, and small-scale team competitions to increase communication, mutual assistance, and a sense of belonging. PE activities should also emphasize fairness, rule awareness, cooperation, and inclusive participation, thereby helping residents from different social backgrounds build more positive social relationships.

Third, PE strategies may be adapted to the needs of different population groups, while recognizing that the subgroup findings were exploratory. For example, group activities may be suitable for residents who benefit from emotional communication and mutual support; team sports and peer-based activities may help expand social networks among young residents; flexible workplace-based or weekend programs may reduce participation barriers for middle-aged adults; and low-intensity collective activities, such as Tai Chi and walking groups, may support social participation among older adults. Future practice should further evaluate whether these differentiated strategies are effective in promoting social interaction and trust-related outcomes.

### 4.5 Limitations

This study has several limitations. First, although the CGSS 2023 provides nationally representative data, its cross-sectional design limits the ability to draw causal inferences. Future studies could use longitudinal data or experimental designs to further examine the causal relationship between PE and social trust.

Second, the dependent variable and the three mediating variables were measured using single-item indicators, it may not fully capture the multidimensional nature of complex psychosocial constructs. Therefore, the findings should be interpreted with caution and should not be generalized to all dimensions of social trust, social interaction, social support, or class identity. Future research could use multidimensional scales to assess these variables more comprehensively.

Third, PE, the mediator variables, and ST were all measured at the same time point using self-reported data from the same survey instrument. This design limits the ability to establish temporal ordering among the variables and may also introduce common method bias. Because all key variables were collected from the same respondents within the same survey context, the observed associations may partly reflect shared measurement characteristics rather than substantive relationships among the constructs. For example, general response tendencies, recall bias, social desirability, or temporary psychological states may have influenced responses across multiple variables, thereby inflating or attenuating the estimated associations and indirect effects. Therefore, the mediation results should be interpreted as potential explanatory pathways rather than causal mechanisms. Future studies should use longitudinal or experimental designs, objective measures of PE, multi-source data, or appropriate tests of common method variance to verify these relationships and reduce measurement bias.

Fourth, because the data are cross-sectional, it is possible that individuals who are more trusting of others are also more inclined to engage in physical activities involving social interaction. Therefore, the findings should be interpreted as associations rather than causal effects, and future longitudinal or experimental studies are needed to clarify this relationship.

Fifth, although this study controlled for several individual, household, and regional characteristics, unobserved factors such as personality traits, health status, community conditions, and psychosocial variables may still influence both PE participation and ST. Therefore, the findings should be interpreted cautiously, and future studies should use more comprehensive measures and research designs to address these potential confounders.

Finally, although subgroup analyses were conducted, formal interaction effects were not examined. Therefore, these findings should be interpreted as exploratory rather than as confirmed evidence of subgroup-specific effects. Future studies should further test these potential moderating roles using interaction terms or formal moderation analyses.

## 5 Conclusion

Drawing on a nationally representative sample from the CGSS 2023, this study examined the association between PE and social trust among Chinese residents. Multiple regression, ordered Probit models, propensity score matching, and mediation analysis were used to assess the robustness of this association and explore potential explanatory pathways. The results showed that PE was positively associated with social trust, indicating that residents who participated in PE tended to report higher levels of trust in others and in broader social relationships. The mediation results suggested that social interaction, perceived social support, and class identity may statistically account for part of this association. Heterogeneity analysis further showed that the positive association was more pronounced among women, young residents, and residents with a university education. Overall, these findings suggest that PE is positively related to social trust and may have potential social relevance beyond its health-related benefits. However, given the cross-sectional and observational nature of the study, these results should be interpreted as associations rather than evidence of causal effects.

## Data Availability

Publicly available datasets were analyzed in this study. This data can be found at http://cgss.ruc.edu.cn/.
